# Supplementary material for: Significant increase of global anomalous moisture uptake feeding landfalling Atmospheric Rivers
Source: Nat Commun. 2020 Oct 8;11:5082. doi: 10.1038/s41467-020-18876-w (PMC7544831; doi:10.1038/s41467-020-18876-w)
Supplement: Supplementary file 1 — Supplementary Information [file 41467_2020_18876_MOESM1_ESM.pdf]

Supplementary Information for

Significant increase of global anomalous moisture uptake feeding landfalling Atmospheric Rivers

Iago Algarra [1], Raquel Nieto[1], Alexandre M. Ramos[2], Jorge Eiras-Barca[1,3], Ricardo M. Trigo[2,4], Luis Gimeno[1*]

[1] Environmental Physics Laboratory (EPhysLab), CIM-UVIGO, Universidad de Vigo, Ourense, 32004, Spain.

[2] Instituto Dom Luiz (IDL), Facultade de Ciências, Universidade de Lisboa, 1749-016 Lisboa, Portugal.

[3] Department of Atmospheric Sciences, University of Illinois at Urbana-Champaign, Urbana-Champaign, IL, USA

[4] Departamento de Meteorologia, Instituto de Geociências, Universidade Federal do Rio de Janeiro, Rio de Janeiro, 21941-916, Brazil

* Luis Gimeno
Email: l.gimeno@uvigo.es

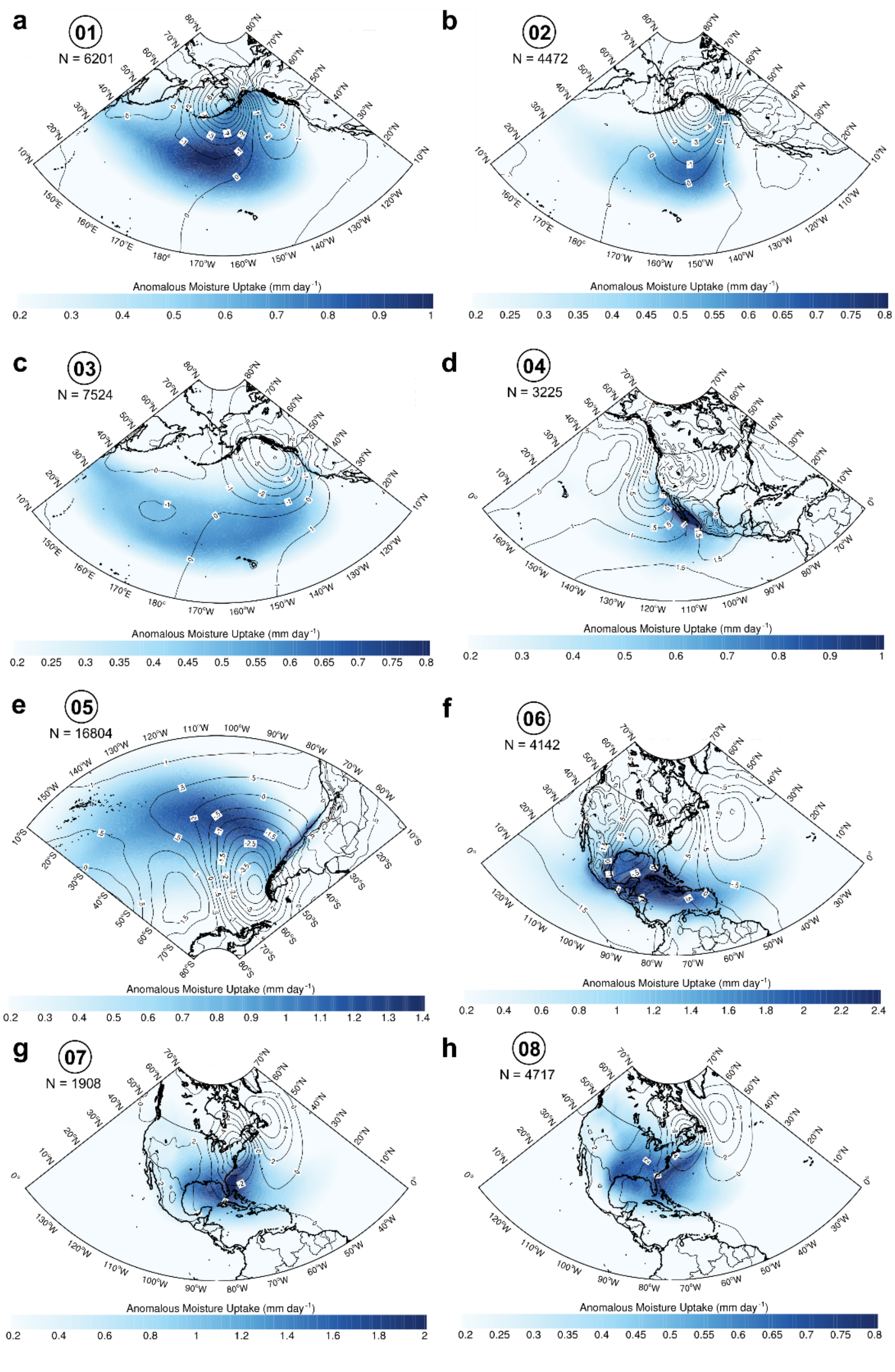

Supplementary Figure 1. Annual anomalous moisture uptake for regions of maximum occurrence of landfalling Atmospheric Rivers from 1980 to 2017. Spatial pattern of Anomalous Moisture Uptake (AMU) for regions 1 to 8 identified in Fig. 1 (in color, mm day^{-1}) and mean sea level pressure anomaly (black isolines, hPa). N denotes the number of landfalling Atmospheric Rivers (LARs) over each region.

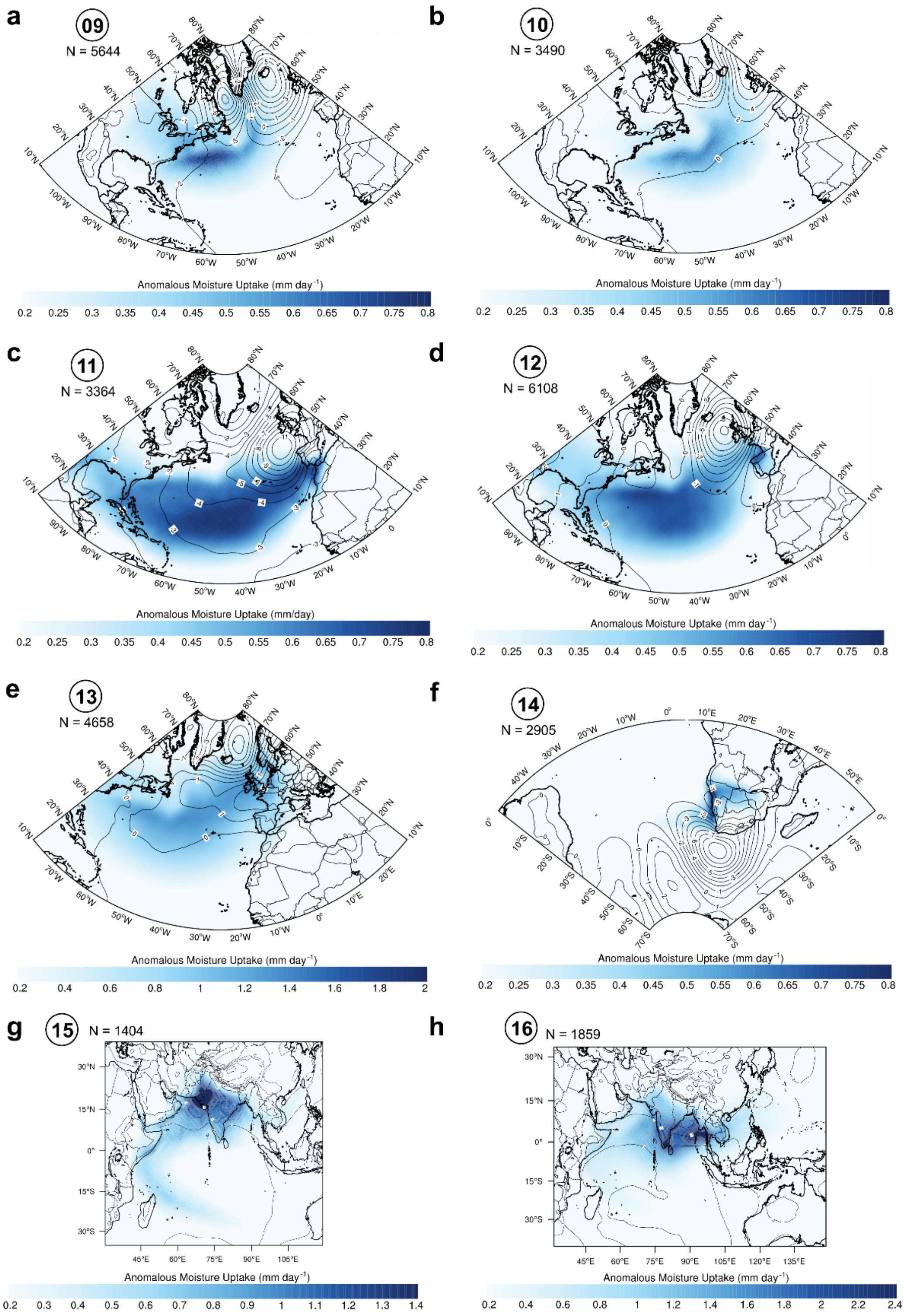

Supplementary Figure 2. As Supplementary Fig. 1 but for landfalling Atmospheric River (LAR) regions from 9 to 16 identified in Fig.1

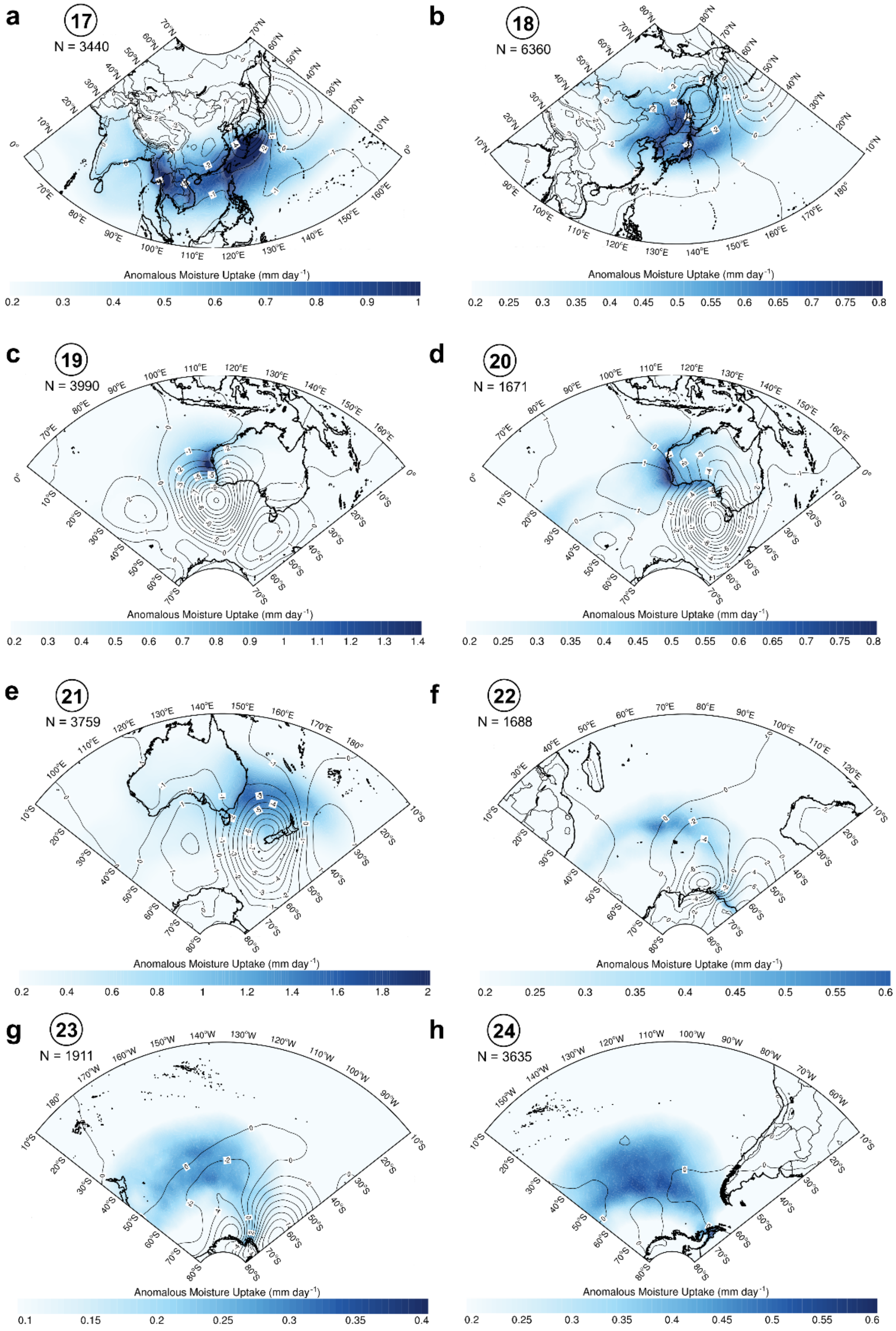

Supplementary Figure 3. As Supplementary Fig. 1 but for landfalling Atmospheric River (LAR) regions from 17 to 24 identified in Fig.1

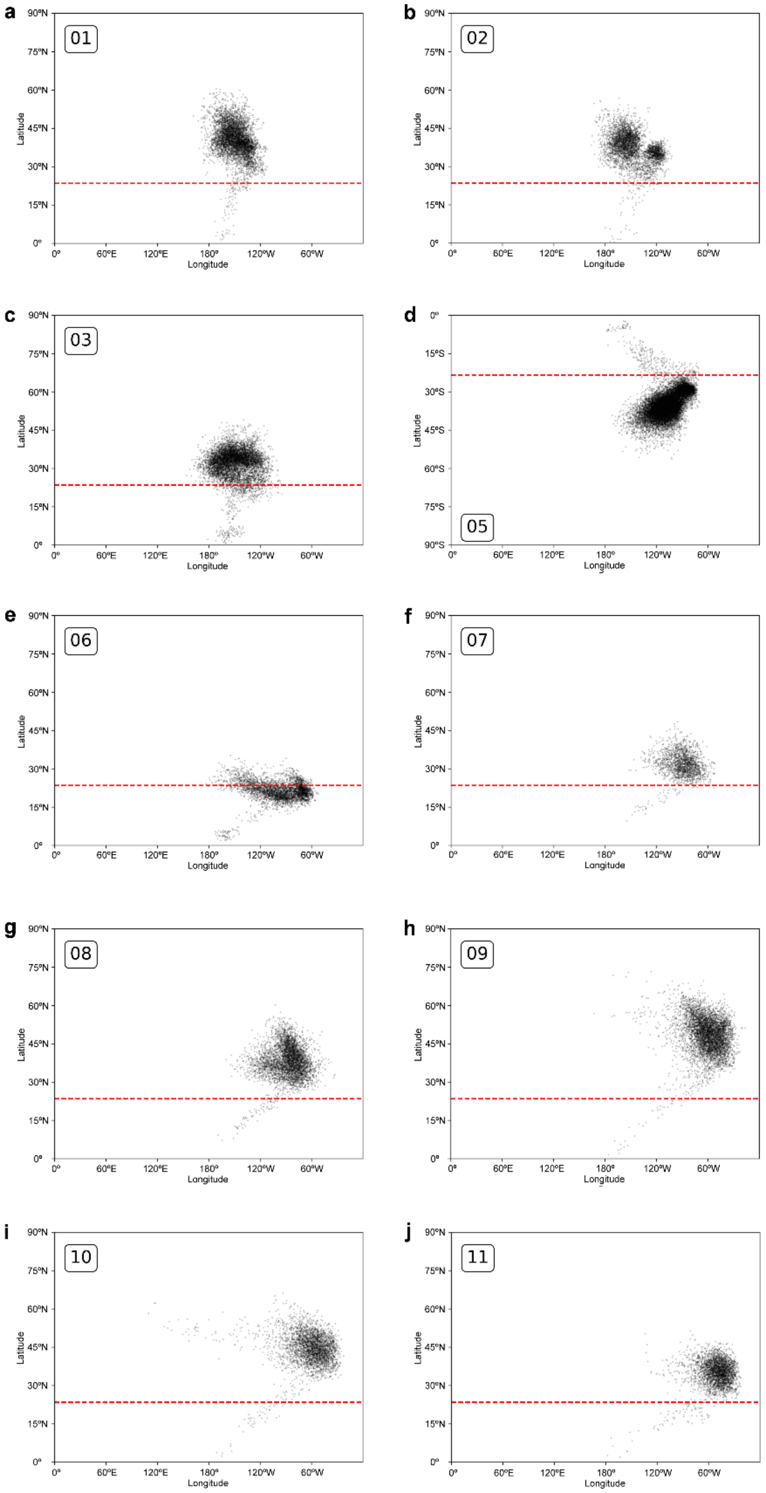

Supplementary Figure 4. Weighted centroids of Anomalous Moisture Uptake for the landfalling Atmospheric Rivers by region. Centroid position (black points) of the area of Anomalous Moisture Uptake (AMU) for each landfalling Atmospheric River (LAR) (defined by the 90 percentile) weighted by each AMU value for region 1 to 11 identified in Fig.1. Red dashed line indicates the Tropic of Cancer (23.43ºN) or Capricorn (23.43ºS).

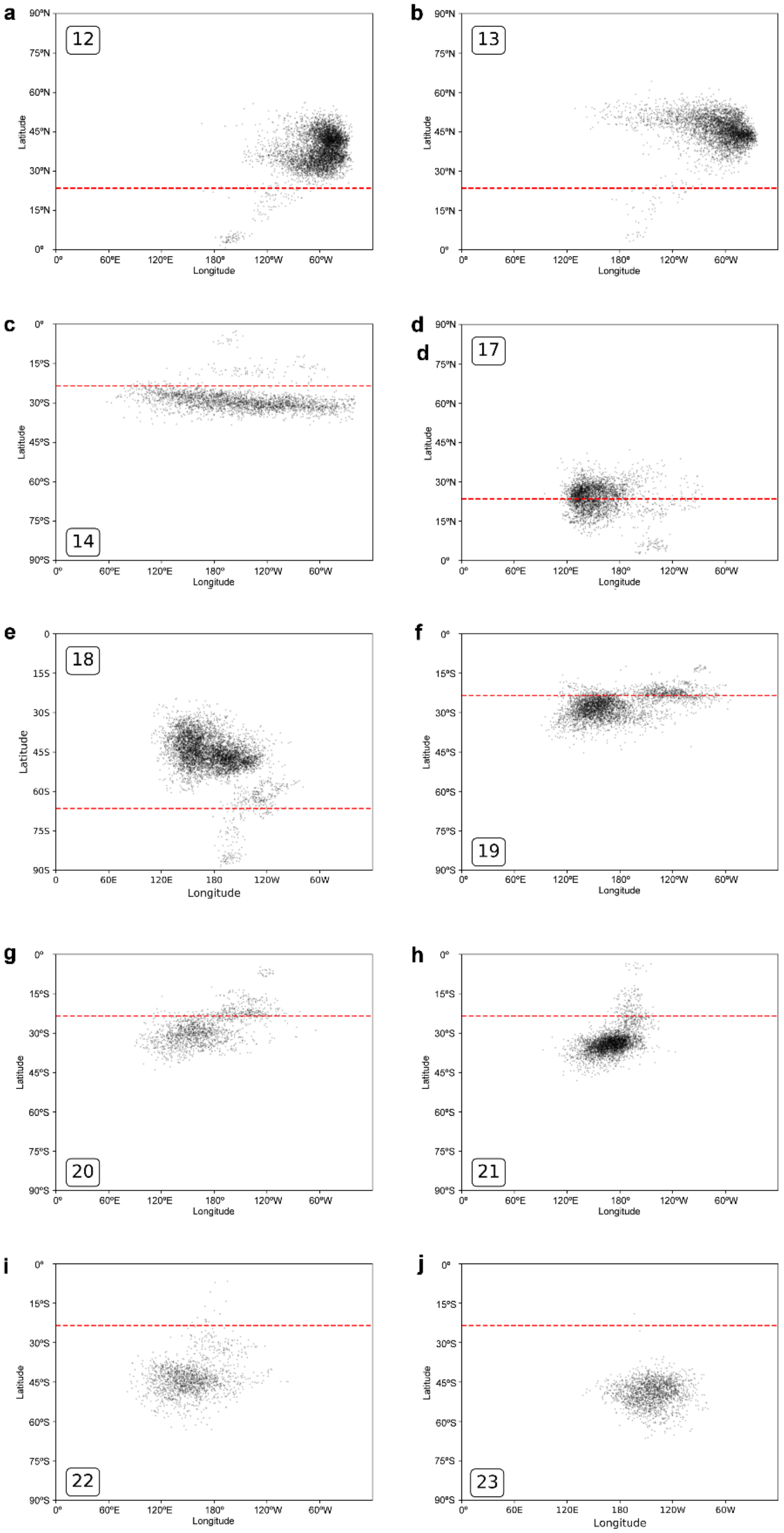

Supplementary Figure 5. As Supplementary Fig. 5 but for landfalling Atmospheric River (LAR) regions 12 to 23.

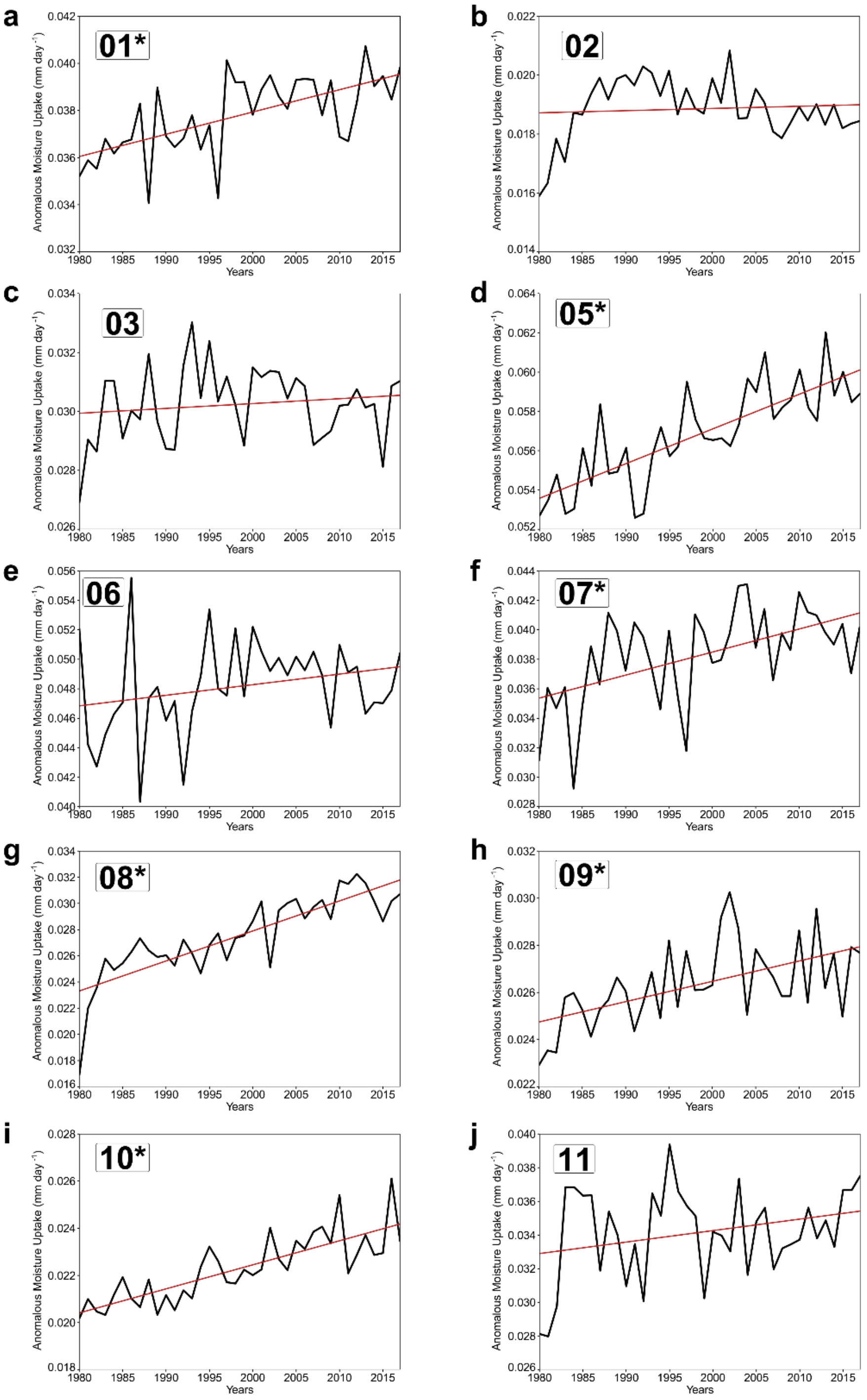

Supplementary Figure 6. Interannual variability of Anomalous Moisture Uptake for each landfalling Atmospheric Rivers region from 1980 to 2017. The red line shows the corresponding linear regression. Box (top left) shows the number of each landfalling Atmospheric River (LAR) region in Fig. 1 (from 1 to 11). The asterisk indicates a significant trend (95%). Trends were calculated using Mann-Kendall test.

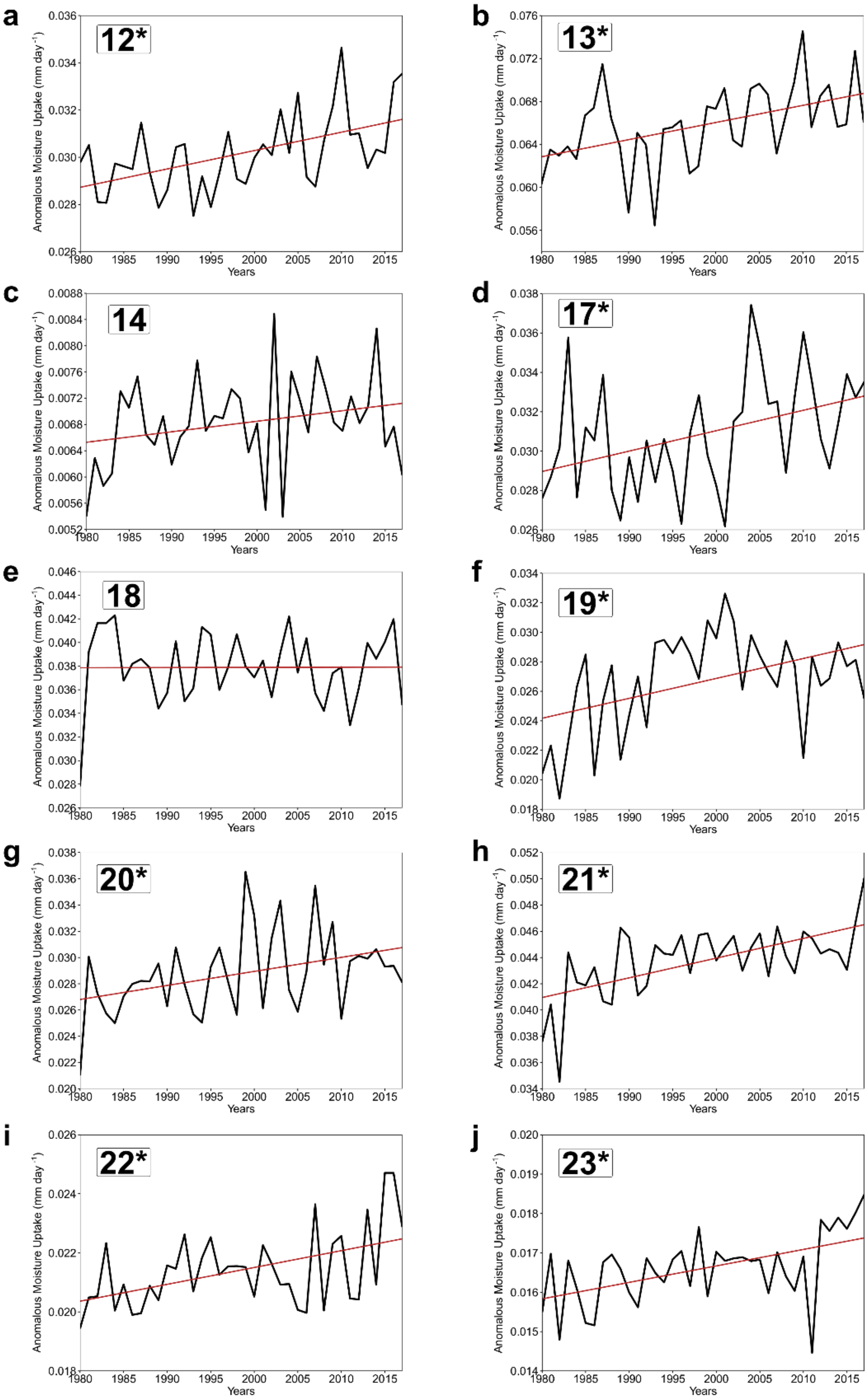

Supplementary Figure 7. As Supplementary Fig. 6 but for landfalling Atmospheric River (LAR) regions 12 to 23.

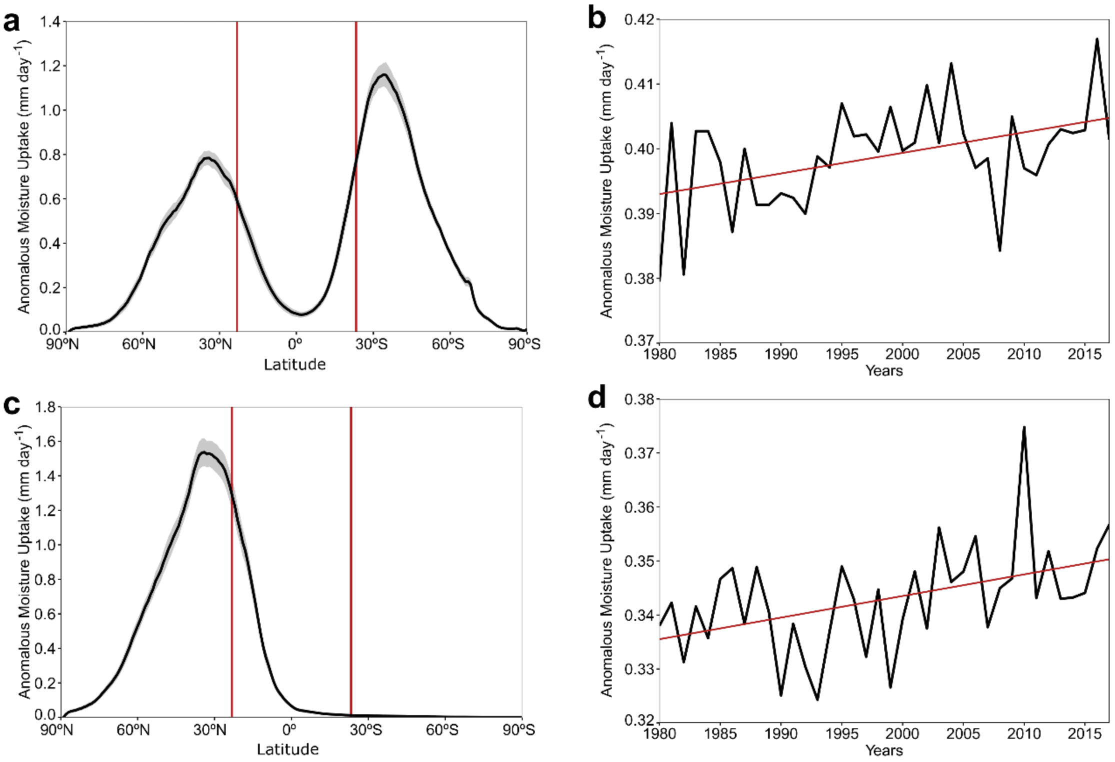

Supplementary Figure 8. **Anomalous Moisture Uptake analysis.** Zonal average of Anomalous Moisture Uptake (AMU) and its interannual variability (shaded grey area) and worldwide annual variability of AMU. (a) and (b) excluding the AMU from North Atlantic Basin. (c) and (d) only for the AMU from North Atlantic Basin. The red lines in (a) and (c) define the region between the Tropics of Cancer (23.43ºN) and Capricorn (23.43ºS). The red line in (b) and (d) shows the corresponding linear regression. The trend was calculated using Mann-Kendall test which is significant in (b) and (d) (95% level of significance).

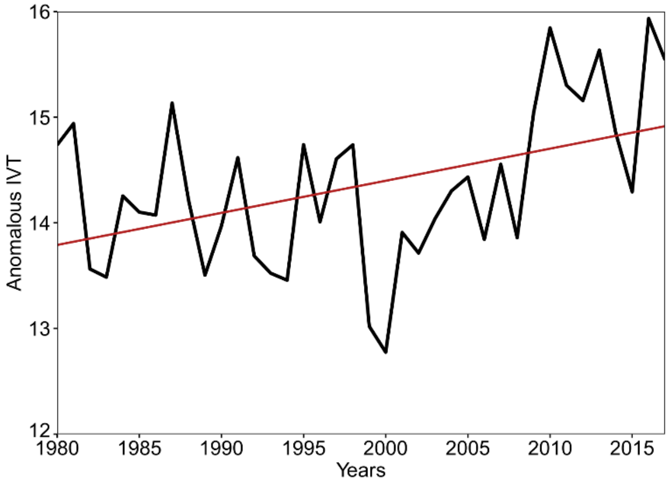

Supplementary Figure 9. Global annual variability of Integrated Vapor Transport anomalies. The red line shows the corresponding linear regression. The trend was calculated using Mann-Kendall test which is significant (95% level of significance).
